# Renal-hepatic-pancreatic dysplasia syndrome (ivemark's syndrome)

**DOI:** 10.1186/1746-1596-2-24

**Published:** 2007-07-02

**Authors:** Mahesha Vankalakunti, Kirti Gupta, Nandita Kakkar, Ashim Das

**Affiliations:** 1 Department of Histopathology, Postgraduate Institute of Medical Education and Research, Chandigarh, India

## Abstract

**Background:**

Renal-Hepatic-Pancreatic dysplasia syndrome described by Ivemark in 1959 constitutes a triad pancreatic fibrosis, renal dysplasia and hepatic dysgenesis.

**Case presentation:**

We describe two unrelated cases of Renal-Hepatic-Pancreatic dysplasia syndrome in stillborn babies. The characteristic microscopic features were present in both the cases. The second case illustrates the unique association lymphangiectasia with Renal-Hepatic-Pancreatic dysplasia syndrome. Both cases are unrelated and there is no history of any consanguineous marriage.

**Conclusion:**

These two cases are unrelated and are rare. In the developmental research, the perinatal autopsy needs to be utilized as a major tool and an Ad hoc committee formation is required to formulate the approach towards syndromic diseases.

## Background

Renal-Hepatic-Pancreatic dysplasia syndrome is a rare sporadic or autosomal recessive disorder characterized by pancreatic fibrosis, renal dysplasia and hepatic dysgenesis. This triad constitutes a "dysplastic sequence" and was first reported by Ivemark et al as "familial dysplasia of kidneys, liver and pancreas" in 1959 [1]. Renal-Hepatic-Pancreatic dysplasia syndrome is uniformly fatal. Most of the deaths occur in their early neonate period. The molecular basis for the defect in the development of epithelial cell of ductular system in renal, hepatic, and pancreas remains unknown. Herein, we describe two stillborn cases with characteristic features of Renal-Hepatic-Pancreatic dysplasia syndrome.

## Case summary 1

A 28-year-old primigravida in the 33^rd ^week of gestation was admitted to the obstetric services with one-day history of abdominal pain and bleeding per vaginum. She did not have any regular antenatal check-up; however, the antenatal period was uneventful. There was no history of consanguinity, drug intake or exposure to radiation in the first trimester. Examination revealed palpable uterus up to xyphisternum, breech presentation and pervaginal bleeding. Fetal heart sounds were audible but feeble. A clinical diagnosis of abruptio placenta was made and labor was induced. A fresh stillborn baby boy weighing 1.4 Kg was delivered. Informed consent was obtained for a complete autopsy.

**Figure 1:**
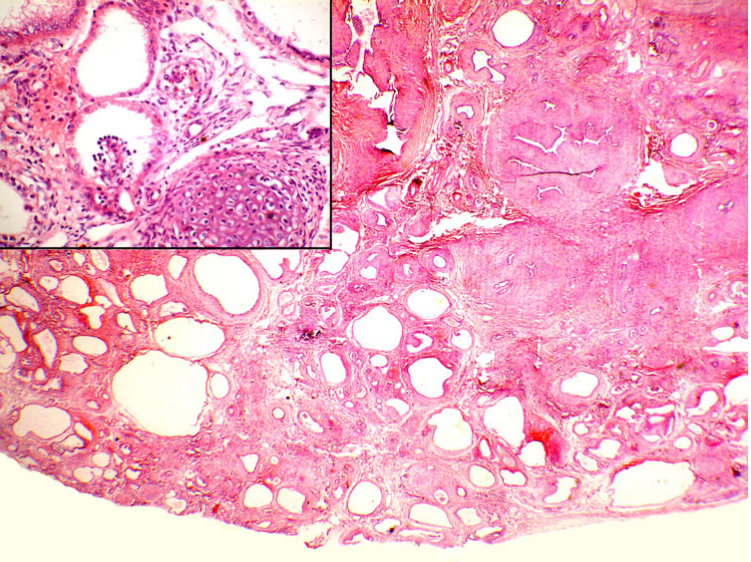
Photomicrography of the kidney shows dilated premature cysts, immature mesenchyme and glomerulus (H&E ×20). Inset showing primitive glomerulus with cuboidal lining epithelium of Bowman's capsule and cartilaginous focus (H&E ×400).

**Figure 2:**
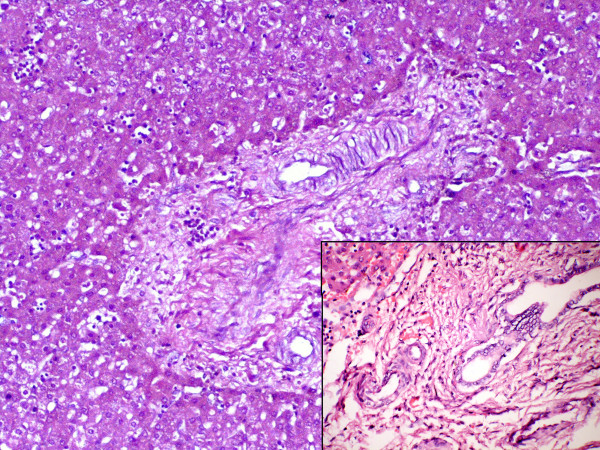
Photomicrography of the liver showing normal hepatic lobule with foci of extramedullary haematopoesis (H&E ×100). Inset shows immature mesenchyme surrounding premature biliary channels (H&E ×400).

On external examination, the baby weighed 1.4 Kg with crown-to-heel length of 41 cm and foot length of 6 cm. Kidneys were mildly enlarged for age and weighed 20 g with normal shape and fetal lobations. The outer surface was predominantly smooth with some areas showing recognizable tiny cysts. On cut section, these cysts were rounded, randomly present in the cortex and medulla ranging in size from 0.2 to 1.2 cm diameter. Pyramids and renal calyces were underdeveloped. Microscopically, there were pathognomonic primitive ducts and the cysts were lined by undifferentiated cells surrounded by immature kind of mesenchyme with cartilagenous islands (Figure 1). Glomeruli were reduced in number and appeared immature. Liver was moderately enlarged, weighed 78 g, and was firm in consistency. Microscopically, the lobular architecture was maintained and portal tracts were round and mildly expanded. The features of dysplasia were seen in the form of primitive ducts in the portal tract lined by bland low cuboidal nondescript cells surrounded by immature mesenchyme (Figure 2). Pancreas was also moderately enlarged for age, weighed 8 g, and was diffusely firm to hard in consistency. On cut section, no cysts were identified. Microscopically, it was densely populated by the proliferation of immature mesenchyme, which at places was encircling dilated ducts lined by similar nondescript cells. Both the lungs and spleen were within normal limits. Thymus displayed features of stress involution.

## Case summary 2

A 24-year-old in the 36^th ^week of gestation was admitted to the obstetric services with loss of fetal movements. Her antenatal check-up at 28 weeks had revealed fetal ascites, scalp edema and cystic kidneys on ultrasound examination. There was no history of consanguinity. A clinical diagnosis of intra-uterine fetal death was suspected secondary to complicated breech presentation, and was taken for emergency lower segment caesarian section. A fresh stillborn baby boy weighing 1.4 Kg was delivered. Informed consent was obtained for a complete autopsy.

**Figure 3:**
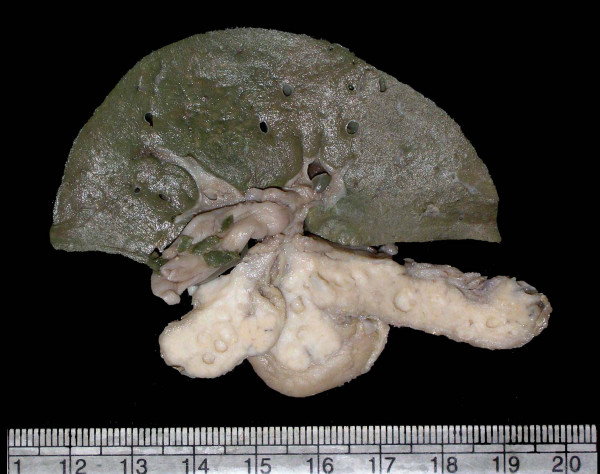
Gross photo of liver and pancreas showing multiple cysts in the latter.

**Figure 4:**
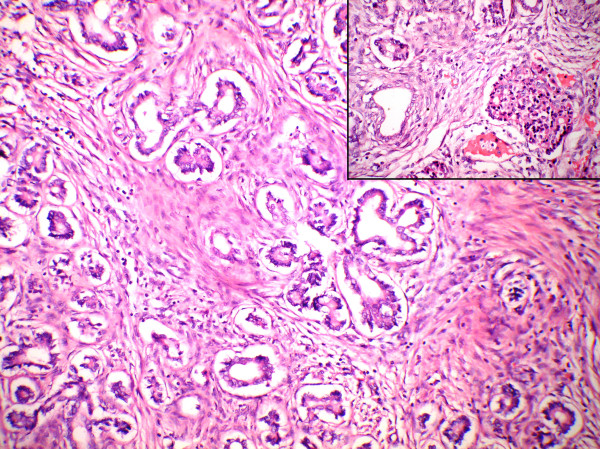
Photomicrography of pancreas shows widely spaced ducts lined by undifferentiated cells with loss of acini (H&E ×100). Inset showing islet cells (H&E ×400).

**Figure 5:**
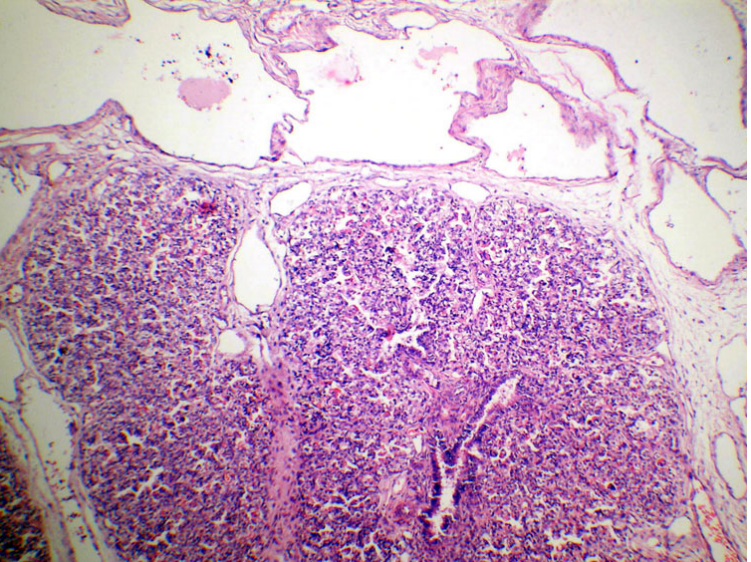
Photomicrography of lung showing varying sized dilated lymphatic channels in the interlobar fissure and pleural tissue (H&E ×100).

At autopsy, the baby weighed 1.4 Kg with crown-to-heel length of 39 cm, foot length of 6 cm. There was congenital talipes equino-varus deformity. Serous effusions were noticed. Kidneys were normal sized and showed multiple tiny cysts measuring 0.2 to 0.8 cm diameter. Microscopy showed features of dysplasia in the form of primitive ducts surrounded by immature mesenchyme. Pancreas was firm and showed cysts grossly, ranging in size from 0.4 to 1.3 cm (Figure 3). Microscopy revealed predominant areas of immature mesenchyme surrounding the cysts which were lined by bland low cuboidal lining (Figure 4). Although reduced in number, foci of islets were present (Figure 4, inset). Liver displayed marked extra-medullary hematopoesis and focal mesenchymal dysplasia in the portal tracts. In addition, lungs showed dilated lymphatic channels in the pleural tissue and also in the inter-lobar and lobular fissure (Figure 5). Blood group of mother was 'O' and Rh positive.

## Discussion

Renal-Hepatic-Pancreatic dysplasia syndrome is a rare sporadic or autosomal recessive disorder characterized by pancreatic fibrosis, renal dysplasia and hepatic dysgenesis. Ivemark et al first reported it as "familial dysplasia of kidneys, liver and pancreas" in 1959 [1]. Since then, this combination of abnormalities has also been named "polycystic dysplasia" and "renal-hepatic-pancreatic dysplasia". This is to avoid confusion with asplenia-cardiac anomaly syndrome, which was reviewed by Ivemark et al and also bears Ivemark's name. Renal dysplasia in Renal-Hepatic-Pancreatic dysplasia syndrome becomes apparent at 16 weeks of gestation and progresses thereafter in severity. It is characterized by disturbance in glomerular differentiation, delay in tubular differentiation and abnormal expression of epithelial markers in glomeruli and tubules [2]. There are reports in the literature in which this syndrome has been picked up by prenatal ultrasonography as early as at 18 weeks gestation [3]. Renal-Hepatic-Pancreatic dysplasia syndrome is uniformly fatal. Most of the deaths occur in their early neonate period. Those who survive the newborn period, the clinical and functional reflection of these include renal insufficiency, chronic jaundice, and insulin-dependent diabetes mellitus.

The average number of perinatal deaths per year in our institute is 306 over the last 5 years, out which 89% undergo autopsy. Both of our cases had the characteristic findings of Renal-Hepatic-Pancreatic dysplasia syndrome i.e., dysplasia involving kidneys, pancreas and liver. The pancreatic dysplasia is more severe in our cases than in those of Ivemark et al. The pelvicalyceal architecture was maintained, thereby ruling out the possibility of obstructive dysplasia as the etiology. In addition, case 2 had external abnormalities like congenital talipes equino-varus deformity, serous effusions, marked extramedullary hematopoesis and lymphangiectasia in the lungs. The association of lymphangiectasia with Renal-Hepatic-Pancreatic dysplasia syndrome is not known.

The two siblings described by Ivemark et al died with renal failure several weeks after birth. These children had bilateral dysplastic kidneys, congenital hepatic fibrosis, and dysplasia of the pancreas without grossly visible cysts [1]. The case described by Strayer et al is a similar variant of Ivemark syndrome in a 1-day-old child [4]. This patient had large pancreatic cyst, multiple large hepatic cysts, congenital hepatic fibrosis, bilateral dysplastic kidneys, and dysplasia of the pancreas.

Cystic changes of these three organs may be an isolated entity or may occur as a final common pathway of response to a variety of developmental disturbances in various polymalformation syndromes. Experimental studies in the pregnant rats have shown that cysts develop in kidney collecting tubules, bile ducts, & pancreatic ducts after administration of a Na/K ATPase blocker, suggesting a common ductular developmental mechanism [5]. Schrick et al have shown in mouse model that absence of ADP-ribosylation factor-like 3 is associated with abnormal epithelial cell proliferation and cyst formation in the renal, hepatic and pancreatic epithelial tubule structures [6].

The polymalformation syndromes include Meckel syndrome, Dandy-Walker cyst, Jeune, trisomy 9, Saldino-Noonan, and Elejalde types of chondrodysplasia, and glutaric aciduria II [7,8]. In Meckel syndrome, extensive deformitives involve the occipital neural tube defects, enlarged cystic kidneys, polydactyly, and other rare sites like liver, heart, and pancreas are seen. Abnormalities of the genital tract have also been described. In addition to the dysplastic kidneys, some cases have been described in which cysts and fibrosis of the liver and even cysts of the pancreas were present [9]. The classical triad of Meckel syndrome described earlier was occipital neural tube defect, enlarged cystic kidneys and polydactyly. The bile duct plate abnormality in the liver is a constant feature [10]. The Meckel syndrome would make an attractive diagnosis in case 2 since it had few of the described deformities of Meckel syndrome. However, the characteristic features of dysplasia in kidney, pancreas and liver and absence of neural defects favour Renal-Hepatic-Pancreatic dysplasia syndrome. Lower cervical neural tube defects occur in Dandy-Walker cyst [11]. The kidneys in autosomal recessive polycystic disease (ARPKD) are enlarged, with fusiform cysts throughout the renal parenchyma running perpendicular to the cortex. No dysplasia is seen in ARPKD. In glutaric aciduria type II, cysts are evident grossly in the liver, and fat infiltration is a consistent feature. Renal lesions are less apparent [12]. Appropriate metabolic tests would help in arriving to the correct diagnosis. The other syndromes have glomerulocystic lesions in the kidney, in excess to the dysplasia.

As highlighted by Bendon RW, there should be formation of Ad hoc committee to facilitate the utilization of the perinatal autopsy in developmental research [13].
